# Implicit bias, neuroscience and reproductive health amid increasing maternal mortality rates among Black birthing women

**DOI:** 10.1002/nop2.1759

**Published:** 2023-06-16

**Authors:** Nia Josiah, Niarah Russell, Lauren DeVaughn, Nadège Dorcelly, Michelle Charles, Hakeem Shoola, Michael Ballard, Diana‐Lyn Baptiste

**Affiliations:** ^1^ Columbia University School of Nursing New York New York USA; ^2^ Substance Abuse and Mental Health Services Administration Rockville Maryland United States; ^3^ Johns Hopkins University School of Nursing Baltimore Maryland USA

Black birthing women in the United States (U.S.) are three to four times (243%) more likely to succumb to a pregnancy‐related death than their White counterparts (Centers for Disease Control and Prevention [CDC], 2022; Hunt, 2021). One of the leading causes of maternal deaths worldwide is preeclampsia. Preeclampsia, a perinatal complication, is a hypertensive disorder of pregnancy experienced at a 60% higher rate in Black birthing women than in White birthing women (Burke, 2020). Black birthing women experience a higher burden of perinatal complications often exacerbated by pre‐existing comorbidities such as hypertension, asthma and gestational diabetes which are less likely to be adequately managed (Burke, 2020). In addition to the influence of pre‐existing comorbidities on perinatal complications, there are other factors impacting maternal health outcomes for Black birthing women. One such factor is repeated exposure and adaptation to chronic stressors such as racism and discrimination which leads to accelerated biological deterioration called ‘weathering,’ and increased allostatic load among Black individuals (Wallace & Harville, 2013). High allostatic load increases vulnerability to stress related diseases, exacerbates pre‐existing comorbidities, and is a contributor to adverse birth outcomes particularly in Black birthing women who are already at a higher risk for severe maternal morbidity (Geronimus, 1992; Howell, 2018; Wallace & Harville, 2013). As a result, simply experiencing pregnancy as a Black woman poses a higher risk for maternal death (Burke, 2020; Howell, 2018).

The CDC (2022) reports multiple factors contributing to aforementioned health disparities, such as barriers to healthcare access. However, Chinn et al. (2021) suggest health inequities experienced by Black women stem from individual, structural and systemic forms of racism. Common behaviours associated with racism include discrimination and implicit bias (Chinn et al., 2021). Evidence‐based literature supports that social determinants of health such as low socioeconomic status, low levels of educational attainment and limited access to healthcare as contributing factors to health disparities. However, for Black birthing women, social determinants of health are not always a causative factor for these poor health outcomes (Gillette‐Pierce et al., 2022). Although maternal health status is multifactorial, racism is an irrefutably significant contributor to the disparities in maternal morbidity and mortality rates. Institutional and structural forms of racism sustain racial inequality across socioeconomic and educational cleavages, ultimately marginalizing ethnic minority groups in their healthcare experiences (Burke, 2020). When Black women receive maternal care, the quality of that care is often below safe standards despite socioeconomic status and educational attainment (Burke, 2020).

## 1 RACISM, IMPLICIT BIAS & NEUROSCIENCE

Racism can be experienced on an individual (interpersonal), communal or systems level. Interpersonal racism manifests as either explicit bias, with deliberate intent, or implicit bias, with unintentional intent (Stevens & Abernethy, 2018). Implicit bias is a covert form of racism that occurs when unconscious thoughts or feelings unknowingly influence one's behaviours towards a specific ethnic group, perpetuating cultural stereotypes (Saluja & Bryant, 2021).

Racial prejudice and stereotypes are constructed in the brain through associative learning, better known as classical fear conditioning (Mattan et al., 2018). The association linking fear to a specific race is formed within a complex network of information exchange in a multitude of regions within the brain (Kubota et al., 2012). Functional magnetic resonance imaging (fMRI) reports this network of information exchange begins in the fusiform gyrus, a region in the brain responsible for racial categorisation based on the identification of familiar facial features (Farmer et al., 2020; Kubota et al., 2012). One associates familiarity within their own ethnic ‘in‐group’ by identifying with facial features that look like their own, also known as in‐group membership. Conversely, fear is associated with other faces that do not look like the in‐group and therefore ‘others’ are categorised as the ‘outgroup,’ also known as outgroup membership. This phenomenon called ‘the own‐race bias,’ is intensified when the in‐group is the majority culture (Amodio & Cikara, 2021; Farmer et al., 2020; Ross, 2020; Stevens & Abernethy, 2018). Research further suggests people are seen in the context of the schemata other people develop about them, thus creating a propensity towards bias and driving behaviour (Ross, 2020). Racially biased in‐group behaviour is pervasive and difficult to alter. It is self‐reinforcing and rooted in early childhood experiences where children first learn to fear other ethnic groups stemming from negative outgroup encounters, and negative images of outgroups perpetuated in streaming media (Stevens & Abernethy, 2018). Fear drives emotion, and the in‐group's racial association to fear affects the in‐group's ability to show empathy for the outgroup, resulting in potential harm (Contreras‐Huerta et al., 2013; Stevens & Abernethy, 2018). The two regions of the brain co‐activated when same race individuals are in pain are the anterior cingulate cortex (ACC) and the anterior insula. Together, these brain regions elicit an empathic neural response (Stevens & Abernethy, 2018). The ACC is responsible for the recognition and regulation of emotion and the anterior insula is responsible for interoceptive and emotional awareness. Studies have found these two brain regions are activated when in‐group members see members of the same group experiencing pain which is associated with affective empathy, a felt sense of pain for in‐group members (Contreras‐Huerta et al., 2013). Juxtaposed, studies have also found when in‐group members observe out‐group members in pain, brain regions such as the ventromedial prefrontal cortex and the dorsomedial prefrontal cortex are more active. These brain regions are associated with cognitive empathy, a logic‐based context lacking a felt sense of pain for out‐group members (Stevens & Abernethy, 2018). These findings offer further insight into the implications and the dangers of holding an implicit racial bias, and the impact of long standing trauma inflicted on out‐groups as a result.

Research suggests underlying implicit racial bias is stored in the amygdala, the ‘primitive’ brain region responsible for perceiving threats, and distinguishing fearful stimuli (Amodio & Cikara, 2021). The amygdala is activated when perceptual information enters the brain as a perceived threat, triggering the activation of the ‘fear response system’ (Kubota et al., 2012). Within the fear response system is the ACC which in addition to recognition and regulation of emotion, monitors for conflicting deliberate intent and implicit bias known as ‘response competition.’ It then uses executive functioning in conjunction with the dorsolateral prefrontal cortex to determine how to impartially respond to said perceived threat. Both brain regions regulate and modulate amygdala activity thus regulating implicit biases and biological fear responses to perceived out‐groups (Kubota et al., 2012; Mattan et al., 2018). The periaqueductal grey and the hypothalamus, trigger a ‘flight or fight response,’ resulting in a nonconscious heightened vigilance (Amodio & Cikara, 2021). This automatic response leads to rapid social categorisation, which has implications for forms of racism such as prejudice, stereotypes and microaggressions (Amodio & Cikara, 2021; Stevens & Abernethy, 2018).

## 2 IMPLICIT BIAS IN REPRODUCTIVE HEALTHCARE

For centuries, Black midwives have played an integral role in improving health outcomes for Black women and their families. Prior to having access to doctors and hospitals, midwives were the primary source of healthcare for Black women in poor rural communities (Baptiste et al., 2021). While hospitalization during labour and delivery is typically a ‘safe’ delivery option, Black birthing women continue to experience the enduring effects of racism in hospital settings. One way to mitigate the devastating consequences of implicit bias is to increase provider concordance (Gillette‐Pierce et al., 2022). Considering the dearth of racially concordant clinicians in the U.S., Black birthing women are less likely to receive obstetric care from a provider that shares their racial identity. As such, implicit bias is a major driver of discriminatory practices and perpetuated in modern‐day clinical environments (Saluja & Bryant, 2021).

Implicit bias silently informs one's decisions and moulds subsequent actions. In turn, the implicit bias that providers experience has particularly costly implications. Even with the purest of intentions, implicit bias can influence providers to discredit pain, dismiss symptoms, and ignore best practices, often silencing voices of Black birthing women (Gillette‐Pierce et al., 2022). Provider bias results in the blatant ignorance of urgent maternal warning signs in Black women.

Consequently, research unearths that non‐Black providers' racial biases, specifically in pain perception, are also associated with racial biases in pain treatment and management resulting in negative maternal health outcomes for Black patient populations (Gillette‐Pierce et al., 2022; Hoffman et al., 2016; Saluja & Bryant, 2021). Providers' inaccurate beliefs about Black patients' perception of pain can lead to dismissive attitudes towards Black birthing women while labouring and delayed responses to warning signs of clinical deterioration (Hoffman et al., 2016; Saluja & Bryant, 2021). Sadly, the belief that Black people are less susceptible to pain is hardly new to the obstetric space (Saluja & Bryant, 2021). In fact, this myth was a foundational tenet upon which the field of gynaecology was developed—Black women were routinely the unanaesthetized test subjects of novel gynaecological surgical interventions performed by physicians in the 19th century (Chinn et al., 2021; Gillette‐Pierce et al., 2022).

## 3 RECOMMENDATIONS

Although literature recommends the integration of diversity in healthcare to foster an atmosphere of concordance and inclusion pedagogy to confront implicitly racist attitudes, diverse workspaces alone are not a sufficient solution (see Table 1). Recommendations include the use of a seven‐step self‐analysis approach, implicit bias tests and implementation of quality improvement and patient safety bundle interventions (Davidson et al., 2022; Howell, 2018; Ross, 2020; Sukhera et al., 2019). It is important to note, these interventions, although a useful starting point, only address one small piece of a larger interconnected problem. Committing the time it takes to unpack and unlearn a lifetime of biases is a crucial part of the solution.

**TABLE 1:** Evidence‐based recommendations for reducing implicit bias in reproductive health.

Seven‐step self‐analysis approach uses unconscious exercises to help identify one's own implicit and racial biases towards Black patient populations. The implicit association test (IAT) is an effective tool in assessing changes in bias when used over time as it measures the medical staff's proclivity for racial partiality. Quality improvement initiatives are fundamental to the continued improvement of maternal care and delivery. Alliance for innovation in maternal health's reduction of peripartum racial/ethnic patient bundle to address implicit bias in reproductive health. Maternal morbidity and mortality are preventable with the implementation of policies, and procedures including haemorrhage and hypertension patient safety bundle interventions. Periodic obstetric emergency simulations will encourage all staff to be prepared when urgent intervention is required. Multidisciplinary drills and trainings strengthen providers both intellectually and kinesthetically. Broadening in‐group contact to include diverse populations, increasing the frequency and quantity of multiracial interactions and curtailing the isolation and discrimination of out‐group persons.

## 4 IMPLICATIONS FOR NURSES

There is an urgent call to action for nurses and other healthcare providers to address the daunting disparities in maternal health and to ensure equitable care across the reproductive health continuum. A heightened awareness of the intersectionality between implicit bias, neuroscience and reproductive health and its deleterious effects on Black birthing women is needed to appropiately address this health inequity. Nurses have an obligation to identify and to address their implicit biases in order to support and to empower patients, while ensuring patient safety.

## 5 CONCLUSION

The birth experience for Black women is largely shaped by historically discriminatory systems and practices, creating a ‘birthing while Black’ phenomenon. Through socially conscious research, education, policy and advocacy, the threat of maternal morbidity and increasing mortality rates can incrementally be eliminated, transforming the overall quality of medical care for Black birthing women and bridging the gap between Black and White pregnancy journeys. Targeting implicit biases is foundational to improving health outcomes for Black birthing women. Active listening, effective communication and establishing trusting relationships are critical towards improving Black maternal health disparities. Further exploration is needed to produce valid assessment tools that detect bias. Authors of this paper call to action for the Black birthing women who are ‘unheard, unseen, disbelieved and dismissed’ to be made visible.

## CONFLICT OF INTEREST STATEMENT

The authors declare no conflict of interest.

## References

[nop21759-bib-0001] Amodio, D. M. , & Cikara, M. (2021). The social neuroscience of prejudice. Annual Review of Psychology, 72, 439–469. 10.1146/annurev-psych-010419-050928 32946320

[nop21759-bib-0002] Baptiste, D. L. , Turner, S. , Josiah, N. , Arscott, J. , Alvarez, C. , Turkson‐Ocran, R. A. , & Hamilton, J. (2021). Hidden figures of nursing: The historical contributions of black nurses and a narrative for those who are unnamed, undocumented and underrepresented. Journal of Advanced Nursing, 77(4), 1627–1632. 10.1111/jan.14791 33565132 PMC9185732

[nop21759-bib-0003] Burke, M. (2020, November). Death of black mother after birth of first child highlights racial disparities in maternal mortality. *NBC News* . Retrieved from https://www.nbcnews.com/news/us‐news/death‐black‐mother‐after‐birth‐first‐child‐highlights‐racial‐disparities‐n1246841

[nop21759-bib-0004] Centers for Disease Control and Prevention (CDC) . (2022). Working together to reduce black maternal mortality. Centers for Disease Control and Prevention Retrieved August 8, 2022, from https://www.cdc.gov/healthequity/features/maternal‐mortality/index.html

[nop21759-bib-0005] Chinn, J. J. , Martin, I. K. , & Redmond, N. (2021). Health equity among black women in the United States. Journal of Women's Health, 30(2), 212–219. 10.1089/jwh.2020.8868 PMC8020496 33237831

[nop21759-bib-0006] Contreras‐Huerta, L. S. , Baker, K. S. , Reynolds, K. J. , Batalha, L. , & Cunnington, R. (2013). Racial bias in neural empathic responses to pain. PloS One, 8(12), e84001. 10.1371/journal.pone.0084001 24376780 PMC3871655

[nop21759-bib-0007] Davidson, C. , Denning, S. , Thorp, K. , Tyer‐Viola, L. , Belfort, M. , Sangi‐Haghpeykar, H. , & Gandhi, M. (2022). Examining the effect of quality improvement initiatives on decreasing racial disparities in maternal morbidity. BMJ Quality & Safety, 31(9), 670–678. 10.1136/bmjqs-2021-014225 35428682

[nop21759-bib-0008] Farmer, H. , Hewstone, M. , Spiegler, O. , Morse, H. , Saifullah, A. , Pan, X. , Fell, B. , Charlesford, J. , & Terbeck, S. (2020). Positive intergroup contact modulates fusiform gyrus activity to black and white faces. Scientific Reports, 10(1), 2700. 10.1038/s41598-020-59633-9 32060333 PMC7021708

[nop21759-bib-0009] Geronimus, A. T. (1992). The weathering hypothesis and the health of African‐American women and infants: evidence and speculations. Ethnicity & Disease, 2(3), 207–221. https://www.jstor.org/stable/45403051 1467758

[nop21759-bib-0010] Gillette‐Pierce, K. T. , Richards‐McDonald, L. , Arscott, J. , Josiah, N. , Duroseau, B. , Jacques, K. , & Baptiste, D. (2022). Factors influencing intrapartum health outcomes among black birthing persons: A discursive paper. Journal of Advanced Nursing. 10.1111/jan.15520 36461641

[nop21759-bib-0011] Hoffman, K. M. , Trawalter, S. , Axt, J. R. , & Oliver, M. N. (2016). Racial bias in pain assessment and treatment recommendations, and false beliefs about biological differences between blacks and whites. Proceedings of the National Academy of Sciences, 113(16), 4296–4301. 10.1073/pnas.1516047113 PMC4843483 27044069

[nop21759-bib-0012] Howell, E. A. (2018). Reducing disparities in severe maternal morbidity and mortality. Clinical Obstetrics and Gynecology, 61(2), 387–399. 10.1097/GRF.0000000000000349 29346121 PMC5915910

[nop21759-bib-0013] Hunt, J. (2021). Maternal mortality among black women in the United States. *Ballard Brief* . https://ballardbrief.byu.edu/issue‐briefs/maternal‐mortality‐among‐black‐women‐in‐the‐united‐states#:~:text=A%202014%E2%80%932016%20national%20study%20on%20pregnancy‐related%20deaths%20in,Black%20women%20243%25%20higher%20than%20for%20White%20women

[nop21759-bib-0014] Kubota, J. T. , Banaji, M. R. , & Phelps, E. A. (2012). The neuroscience of race. Nature neuroscience, 15(7), 940–948. 10.1038/nn.3136. 22735516 PMC3864590

[nop21759-bib-0015] Mattan, B. D. , Wei, K. Y. , Cloutier, J. , & Kubota, J. T. (2018). The social neuroscience of race‐based and status‐based prejudice. Current Opinion in Psychology, 24, 27–34. 10.1016/j.copsyc.2018.04.010 29730465

[nop21759-bib-0016] Ross, H. J. (2020). Everyday bias: Identifying and navigating unconscious judgments in our daily lives. Rowman & Littlefield.

[nop21759-bib-0017] Saluja, B. , & Bryant, Z. (2021). How implicit bias contributes to racial disparities in maternal morbidity and mortality in the United States. Journal of Women's Health, 30(2), 270–273. 10.1089/jwh.2020.8874 33237843

[nop21759-bib-0018] Stevens, F. L. , & Abernethy, A. D. (2018). Neuroscience and racism: The power of groups for overcoming implicit bias. International Journal of Group Psychotherapy, 68(4), 584. 10.1080/00207284.2017.1315583 38449159

[nop21759-bib-0019] Sukhera, J. , Wodzinski, M. , Rehman, M. , & Gonzalez, C. M. (2019). The implicit association test in health professions education: A meta‐narrative review. Perspectives on Medical Education, 8(5), 267–275. 10.1007/s40037-019-00533-8 31535290 PMC6820611

[nop21759-bib-0020] Wallace, M. E. , & Harville, E. W. (2013). Allostatic load and birth outcomes among white and black women in New Orleans. Maternal and Child Health Journal, 17(6), 1025–1029. 10.1007/s10995-012-1083-y 22833335 PMC3504172

